# Patient Safety Requires a New Way to Publish Clinical Trials

**DOI:** 10.1371/journal.pctr.0010006

**Published:** 2006-05-19

**Authors:** Richard Smith, Ian Roberts

The way medical journals publish the results of clinical trials has become a serious threat to public health. You may find this assertion shocking and counterintuitive, but we hope that by the end of this short article you will agree and will join us in arguing for the better way of making medical information publicly available that we outline.

## Journals Are Publishing Partial and Biased Reports from Trials

The publication of a clinical trial marks the birth of new medical knowledge, and medical editors are the midwives. Although most editors would like to meet expectant researchers shortly after a clinical trial's conception (or even before), to find out who the parents are and to ensure that the trial receives high-quality antenatal care, more often than not labouring researchers arrive at their offices heavily pregnant with results that require immediate, fast-track delivery. Some trials are deposited on the editor's doorstep, so that it is hard to tell who the parents are. Unfortunately, many trialists have become eugenicists, highly adept in the selective breeding of favourable results [1]. They do this to serve the masters who pay them, often the pharmaceutical industry. Their masters find favourable results useful for marketing [2]; the trialists have their pockets lined and their careers advanced. The editors have newsworthy trials to publish, and the owners of the journal enjoy the substantial profits that come from selling reprints of the trials. The losers are the trial participants whose contribution to research is wasted, the patients who must swallow the drugs despite the distorted evidence, and the public who must pay for the drugs [3].

## A Better Way

The new model we propose would start with posting a systematic review of the existing trial evidence on the Web to show what is already known about the effectiveness of a particular treatment and what further research is needed. If there is uncertainty about the effectiveness of the treatment, such that a further trial is needed, a new trial would be registered and the trial protocol would also be posted on the Web. Everybody with any involvement in the trial would be listed with their contribution explained, abolishing the current need for “paternity testing” of published trials. At any point, observers—be they patients, researchers, clinicians, editors, or anyone else—would be able to comment online about the interpretation of the systematic review's data, the importance of the trial question, or the reliability of its methods.

In this new world, there would be no investigator-driven and thus potentially biased post hoc analysis, no discussion sections and thus no spinning of the results, and no peer review of trial reports.

The statistical analysis would be pre-specified by uploading the programming code (in Stata or SAS, for instance) and a specification for the final dataset. The protocol would include detailed specifications of any subgroup analyses, giving their biological rationale and the anticipated direction of effects [4]. The analysis could also incorporate routine statistical tests to check for the presence of data fabrication and falsification [5,6]. When data collection in the new trial is completed, the entire dataset would be uploaded and the analyses would be run. There would be no investigator commentary on the trial data. The systematic review would be updated to include the new trial.

*PLoS Clinical Trials* is a step in the direction we are proposing, and may perhaps in the long run form a platform for the full system. The role of traditional medical journals would be to comment and debate on all the stages of the process rather than publish the trial results [2]. For practitioners, they might also report on systematic reviews—because, it is unlikely that many practitioners will access full datasets on the Web. They should report on reviews not individual trials to avoid presenting their readers with only part of the evidence. (There will sometimes be systematic reviews that include only one trial.) It will be important, however, for journals to do all they can to avoid bias in their reporting and for practitioners to understand that no matter how hard journals try to be unbiased they will never succeed entirely.

A new model is needed because the current one is in tatters: clinical trial results are being manipulated. What matters is the totality of the relevant trial evidence. By publishing individual clinical trials ad hoc, the medical journals provide a mechanism that can be subverted by funding bodies and researchers with an interest in getting particular trial results.

**Box 1. Problems with the Present System of Publishing Trials**

• There is too much emphasis on the results of individual trials rather than the totality of the evidence

• Trials are conducted and published without a systematic review of existing evidence, meaning that trials are conducted unnecessarily or don't address the most important questions

• Trials often deviate considerably from protocols, but readers of trial results don't know this

• Results are selectively published with positive results emphasised or published more than once, and negative results are ignored

• Full results, including all side effects, are not published

• Published trial results are usually favourable to sponsors because of a wide variety of methods of manipulation

• Methods of analysis are not clear, and post hoc analyses are not presented as such

• Authorship of trials is unclear

• Introduction and discussion sections are often little more than spin

• Trials favourable to sponsors are published in major journals, and unfavourable trials are not published at all or are published in minor journals

## The Current Model of Publishing Trials Is in Tatters

The most common eugenic techniques are selective reporting and the creative use of probability [1]^.^ There may be late termination of trials with unfavourable results, such that they are withheld from the publication process, or else unfavourable findings may be surgically excised from the trial publication [3,7–9]. On the other hand, trials with favourable results may be published many times (cloning), and then intensively marketed to ensure they are noticed. Methodologists believe these are the most common and the most important forms of misconduct in clinical trials [1]. Nevertheless, although editors will not hesitate to name and shame if they suspect data falsification, which is comparatively rare, they seem to be relaxed about selective reporting. They pounce on the shoplifting bag ladies but turn a blind eye to white collar crime.

Posting trial protocols on the Web would allow open debate about the importance, relevance, and quality of the trial [10,11]. Indeed, proper peer review of trial questions and methods would be a better way of improving medical research than peer reviewing trial reports, especially when it is often impossible to tell what information has been omitted. Too many trials cover issues that matter to drug companies (showing that the 24th beta-blocker is better in some way than the 23rd) rather than answering questions that matter to patients. When the trial is over, the full dataset would be uploaded to accompany the trial protocol. Pre-programmed analyses would prevent deviation from the protocol, suppression of results, and any undue emphasis on post hoc subgroup analyses [12,13]. Posting full datasets could allow quicker identification of adverse effects and more critical analysis of the raw data, which may reduce the chances of fraud.

In this new world, there would be no investigator-driven and thus potentially biased post hoc analysis, no discussion sections and thus no spinning of the results, and no peer review of trial reports since all this would have been done at the protocol stage. The discussion section of a scientific paper typically has five functions: (1) to state the principal findings, (2) to identify strengths and weaknesses of the study, (3) to identify strengths and weaknesses in relation to other studies (there is good evidence that most trials fail to do this), (4) to state the meaning of the study, (5) and to identify unanswered questions for future research (M. Clarke, S. Hopewell, I. Chalmers, unpublished communication) [14–16]. The first is best summarised numerically in the point estimates and confidence intervals; the second is determined by trial design, which is evident from the protocol; and the remainder are more appropriate in the context of the relevant systematic review. The discussion section of a clinical trial is therefore redundant.

Posting on the Web the updated systematic review would be more useful for patients and clinicians and would avoid the hyping of single trials. Ending the publication of trials in journals would reduce the manipulation, spin, and hype that are now pervasive in medical publishing. Restricting journals to comment, debate, and digestion would make them more readable and more useful to patients and doctors alike.

**Box 2. Our Proposed New System**

• A systematic review is posted on the Web

• If a new trial is needed, it is registered and a full protocol is devised in light of the systematic review and posted on the Web

• Anybody can comment online on the interpretation of the systematic review data, the importance of the trial question, or the reliability of its methods

• The statistical analysis would be pre-specified and pre-programmed

• When data collection is completed, the entire dataset would be uploaded and the analyses run

• There would be no investigator commentary on the trial data

• The systematic review would be updated to include the new trial

• Journals would not publish trials but rather commentaries and reports on systematic reviews

## Vested Interests Are a Barrier to Change

These proposals should improve the quantity and quality of information on the effectiveness and safety of medical treatments, and for this reason, they should be welcomed. Not everyone will agree. Drug companies are nervous about posting protocols because they claim it could give away competitive information. But if all companies have to post their protocols, there would be no comparative disadvantage. It is the marketing departments of companies that will be most likely to object to the new way of posting protocols and results. Hype will be made much more difficult, and market advantage will be hard to achieve unless companies have products that truly are superior. Medical journals love the loot and prestige that goes with publishing clinical trials, and many would disappear if trials were no longer published. However, the writing is already on the wall for traditional clinical trial publication. Citation analysis already shows that systematic reviews and meta-analyses receive more citations than any other study design [17]. This trend is likely to continue. Researchers might be reluctant to make available “their” raw data out of fear that this will provide material for their intellectual “competitors.” On the other hand, if they want to maintain the respect of the general public, in the context of several widely publicised cases of medical fraud, they will have to make datasets available for statistical scrutiny [18].

All change tends to be resisted, and we've outlined reasons why drug companies, researchers, and journals will all oppose what we propose, and these are the voices that are heard most loudly in any debate over trials. Nevertheless, we believe that our proposal will eventually be implemented. The “platform will begin to burn” as more evidence emerges of patients being harmed by the manipulation of trial results. Governments and others who must pay for hyped drugs will also join the debate as they better understand how trial evidence is being manipulated. Eventually, governments will mandate new ways to make the results of trials available, just as they are already mandating the registering of trials.
